# Effects of high-fat diet and treadmill running on the hypothalamic Kiss-1–GPR54 signaling pathway in male growing rats

**DOI:** 10.1007/s42000-022-00389-4

**Published:** 2022-08-24

**Authors:** Rui Xu, Junpeng Feng, Chunyu Liang, Ge Song, Yi Yan

**Affiliations:** 1 Beijing Sport University, Beijing, China; 2 Nanjing Sport Institute, Nanjing, China

**Keywords:** Kiss-1-GPR54, High-fat diet, Exercise, Growing period, HPG

## Abstract

**Background:**

Kiss-1 neuron, one of the metabolic sensors in the hypothalamus, is necessary for puberty initiation. It acts through G protein-coupled receptor, known as GPR54. In this study, the mechanism of the hypothalamic Kiss-1–GPR54 signaling pathway in a high-fat diet and exercise was investigated in growing male rats.

**Methods:**

A total of 135 3-week-old male weaned rats were kept on a high-fat diet (HFD) and exercise (60–70% $\dot{\mathrm V}{\mathrm O}_{2\max}$, 1 h/day, 5 days/week). They were randomly divided, as follows: control group (C); normal diet + exercise group (CE); HFD group (H); and HFD + exercise group (HE). Hypothalamus, testis, and serum samples of each group were collected on postnatal day (PND) 21 (early childhood), 43 (puberty), and 56 (maturity). Immunofluorescence, quantitative real-time PCR, hematoxylin and eosin staining, and chemiluminescent immunoassays were used in the study. ANOVA was used to analyze the effects of age (PNDs 21, 43, and 56), exercise (exercise and sedentariness), and diet (high-fat and normal) on the biological indices of rats.

**Results:**

mRNA and protein expression of Kiss-1 and GPR54 in the hypothalamus gradually increased along with growth and peaked at PND 43, while those in serum testosterone increased and peaked at PND 56. The high-fat diet increased the expression of the Kiss-1–GPR54 system in the hypothalamus, whereas the serum testosterone decreased during different stages of growth. Exercise decreased the expression of Kiss-1 at PND 56 and increased it at PND 43. Meanwhile, it decreased testosterone and the deposition of lipid droplets in the testis at all ages of development.

**Conclusions:**

The expression of Kiss-1–GPR54 in male rats showed fluctuating changes during growth and development. The high-fat diet was able to upregulate the expression of the Kiss-1–GPR54 system in the hypothalamus. The exercise was able to correct the adverse effect of the high-fat diet on the Kiss-1–GPR54 signaling pathway in the hypothalamus and the function of the hypothalamic-pituitary–gonadal (HPG) axis, but had age-specific effects on the male rats’ development.

**Supplementary Information:**

The online version contains supplementary material available at 10.1007/s42000-022-00389-4.

## Introduction

Puberty is an important developmental stage that requires maturation of the reproductive neuroendocrine axis and subsequent initiation of high-frequency, episodic release of gonadotropin-releasing hormone (GnRH) [1]. Overfeeding or a high-fat diet can induce precocious puberty in boys and girls [2], correlating with body weight and body mass index. Childhood obesity disrupts the pubertal onset of children and further lowers their future quality of life.

Kiss-1 and its receptor gene GPR54 in the hypothalamus, especially in the arcuate nucleus (ARC), play a key role in the initiation of puberty [3]. The Kiss-1–GPR54 system could activate the phospholipase-C (PLC)-β pathway [4, 5], cause depolarization of hypothalamic GnRH neurons, and then stimulate luteinizing hormone (LH) and follicle-stimulating hormone (FSH) secretion. Energy states could affect the expression of Kiss-1 in the hypothalamus [3, 6]. In rat studies, the expression of hypothalamus Kiss-1 increased significantly in high-fat-induced obese prepubertal and pubertal rats [7, 8], whereas the expression of Kiss-1 and the secretion of LH and FSH from the hypothalamic-connected pituitary gland clearly decreased in fasting female rats [9]. Negative energy balance due to undernutrition can induce puberty delay by suppressing ARC Kiss-1 neurons, and positive energetic cues trigger puberty onset via an increase of Kiss-1 in the hypothalamus and GnRH secretion [10].

Exercise has attracted much attention because of its ability to reduce energy production and decrease body-fat accumulation. Recent studies have reported that exercise could be an effective measure to prevent childhood obesity [11] and central precocious puberty [12]. The Kiss-1–GPR54 system plays an important role in puberty initiation control. However, whether exercise could alter high-fat-diet-induced precocious puberty through the Kiss-1–GPR54 signaling pathway in the hypothalamus has yet to be determined. In the above study, most cases were focused on the relationship between the Kiss-1-GPR54 system and energy status in females [13–15]. In fact, as yet fragmentary evidence suggested that the Kiss-1-GPR54 system may also be affected by energy status and metabolic homeostasis in males [16, 17]. Moreover, the effect of exercise intervention on the Kiss-1-GPR54 system in male rats on a high-fat diet at different developmental stages remains virtually unexplored to date. Thus, in this study, the effects of high-fat diet and moderate-intensity treadmill training on the hypothalamic Kiss-1–GPR54 signaling pathway as well as their effects on the reproductive organs of male growing rats were examined.

## Materials and methods

### Animals

The animal experiments were approved by the Animal Welfare Ethics Committee of Beijing Sport University (approval number: 2016021A). A total of 135 male Sprague–Dawley weaned rats on PND 21 (58.5 ± 2.7 g) were purchased from Beijing Vital River Laboratory Animal Technology Company Limited (license number: SCXK 2012–0001; Beijing, China). The rats were kept in a controlled room temperature (25 °C ± 0.5 °C) with a 12-h light/12-h dark cycle. Five rats were housed per cage, with access to food and water ad libitum.

### Experimental design

The animals were randomly divided into four groups on PND 21, as follows: control group (C, *n* = 45), normal diet with exercise group (CE, *n* = 30), high-fat-diet control group (HC, *n* = 30), and high-fat-diet with exercise group (HE, *n* = 30). The C and CE were fed a normal diet (20% protein, 70% carbohydrate, 10% fat, energy density = 3.85 kcal/g; product ID: D12450B). The HC and HE were fed a high-fat diet (20% protein, 35% carbohydrate, 45% fat, energy density = 4.73 kcal/g; product ID: D12451). All food products were purchased from Beijing HFK Bioscience, China.

#### Exercise training

The rats in the CE and HE groups were trained with an intensity of 60–70% of the maximum oxygen uptake ($\dot{\mathrm V}{\mathrm O}_{2\max}$) for 60 min/day, 5 days per week (rat treadmill, DSPT-208; Hangzhou Segment Manufacturing, China). This range was chosen because 60–70% of $\dot{\mathrm V}{\mathrm O}_{2\max}$ represents moderate-intensity exercise: fats are mainly oxidized at aerobic exercise intensities [18]. In the first 3 days of adaptive training (speed = 10 m/min, 0° incline), the durations were 10, 20, and 30 min. After the adaption period, the incline was raised to 10° for the duration of the training period. The treadmill training process was divided into three stages, as follows: (1) warm-up period (5 min), (2) training period (50 min), and (3) cool-down period (5 min). During the warm-up and cool-down periods, the running speed was set at 10 m/min, while the running speed during the training period was 60–70% $\dot{\mathrm V}{\mathrm O}_{2\max}$ (V = 12–17 m/min). The treadmill speed during the training period was gradually increased as capacity improved, as determined in accordance with the corresponding speeds of 100% $\dot{\mathrm V}{\mathrm O}_{2\max}$ of rats at different ages.

#### Tissue collection

**Fig. 1:**
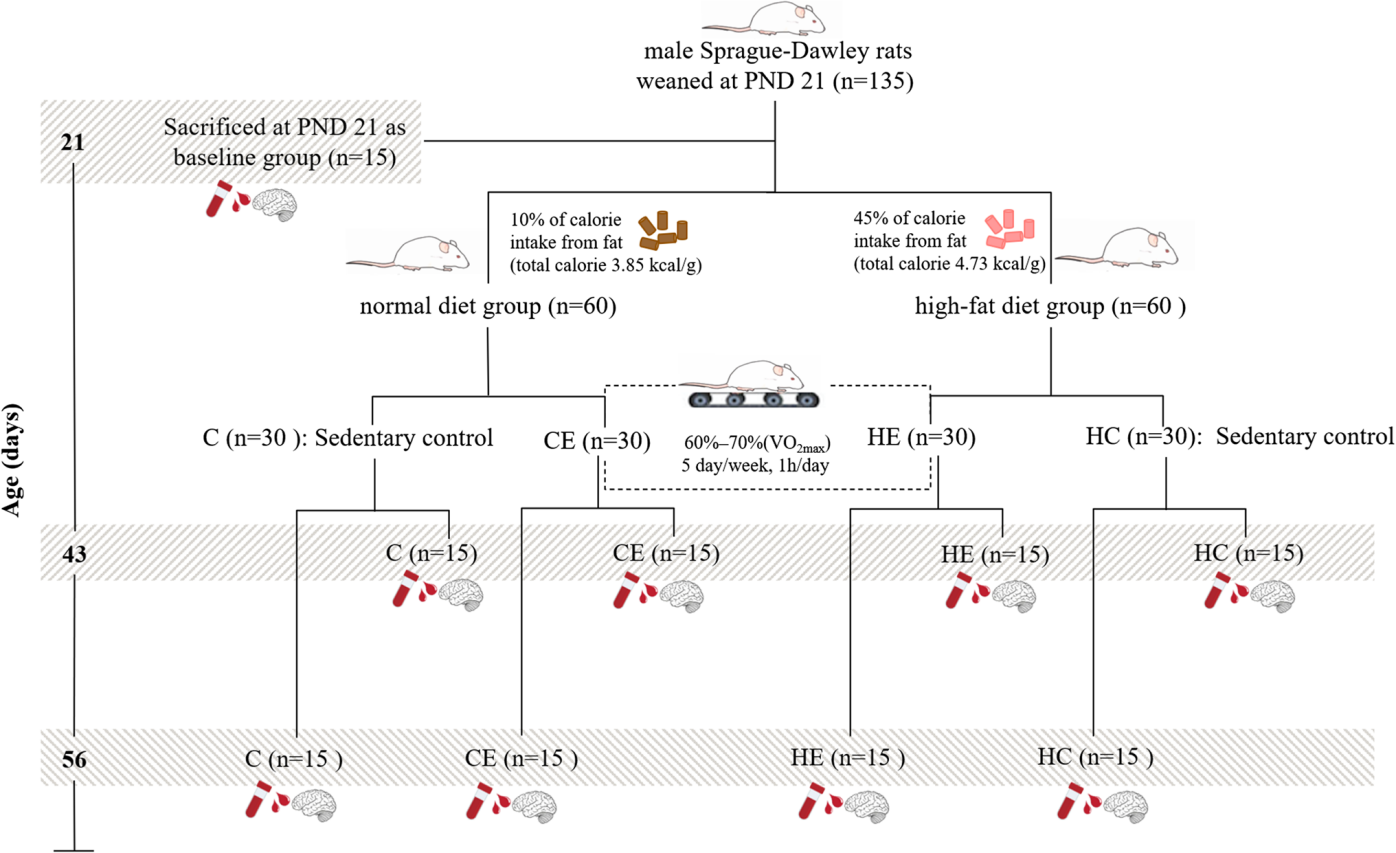
Experimental flow

The rats were sacrificed at 9:00–10:00 AM on the day and at the age of PNDs 43 and 56 from both groups (*n* = 15 for each group and time point). An additional time point on PND 21 (*n* = 15) at the beginning of the study was established as a baseline. All rats were fasted 6 h before being sacrificed and the rats of the exercise groups were sacrificed 48 h after the last exercise training. The experimental flow of the study is presented in Fig. 1.

A total of three rats in each group received thoracotomy, perfused through the left ventricle with saline, followed by 4% paraformaldehyde after deep anesthetization. The brains were removed and immersed in 4% paraformaldehyde for a 24-h post-fixation period. Then, they were placed in 75% ethanol, dehydrated in an ascending series of gradient ethanol cleared in xylene, and embedded in paraffin for subsequent immunofluorescence.

Another 12 rats of each group were rapidly anesthetized with 2% sodium pentobarbital (0.25 Ml/100 g body weight). The abdominal aortic blood was centrifuged to collect serum for the serum hormonal test. The hypothalamus and one side of the testes were frozen immediately in liquid nitrogen and stored at − 80 °C for quantitative real-time PCR (qRT-PCR) after euthanasia. The contralateral testes were immediately removed and immersed in 4% paraformaldehyde for a 24-h post-fixation period, dehydrated in an ascending series of gradient ethanol, and then embedded in paraffin for subsequent hematoxylin and eosin (HE) staining. All the samples that not were immediately used were stored at −-80 °C.

### HE staining

Testes tissues were excised and fixed overnight in 4% paraformaldehyde and embedded in paraffin. Sections (4 µm thick) were cut and subjected to HE staining for histological analysis. Digital images (200 ×) were captured from five random fields per section by using a Leica microscope.

### Hormonal assays

The testosterone contents in serum samples were measured via chemiluminescent immunoassays (CLIAs) by using a Beckman Coulter UniCel DxI 800 Access Immunoassay System (Beckman Coulter, USA).

### qRT-PCR analysis

Total RNA was extracted from the hypothalamus by using an Animal Tissue RNA Extraction Kit (Tiangen, China) in accordance with the manufacturer’s protocol. RNA was reverse transcribed using the Toyobo ReverTra Ace qPCR RT kit (Toyobo, Japan). Then, cDNA was stored at − 20 °C until further use. Subsequently, qRT-PCR was performed using a Bio-Rad iCycler Detection System and a Quantitect SYBR Green PCR kit (TaKaRa, Japan). The primer sequences were as follows: Kiss-1 (F: ATGATCTCGCTGGCTTCTTG, R: CTGTGGGTTCAGGGTTCAC), GPR54 (F: CCACATGTGCCACTTTGACA, R: AACCCACCCAGATGCTAAGG), PLC (F: CTGGATGAGAACAGCCCACT, R: CATGCTGATGGAGAAGACGA), and GnRH (F: CCGCTGTTGTTCTGTTGACT, R: GCAGATCCCTAAGAGGTGAA). Standard curves were generated, and β-actin was used as a housekeeping gene to quantify the abundance of cDNA in each sample. The qRT-PCR cycling parameters were as follows: 95 °C for 15 min and 40 cycles of 94 °C for 15 s, 60 °C for 30 s, and 72 °C for 30 s. Data collection was performed at the 72 °C extension phase. A dissociation curve was drawn after each run to ensure the presence of a single product. Data were collected from the threshold values by using the automatic function of the Bio-Rad MyIQ software. All samples were run in duplicate. The data for Kiss-1, GPR54, PLC, and GnRH mRNA were normalized to those of β-actin.

### Immunofluorescence double labeling and confocal microscopy

Hypothalamic tissue blocks were dehydrated in an ascending series of ethanol, cleared in xylene, and embedded in paraffin. Immunofluorescent staining was used to observe the expression of Kiss-1 and GPR54 proteins in the ARC of each group. Hypothalamic sections (10 μm in thickness) were deparaffinized, and antigen retrieval was performed with 0.01 M of sodium citrate buffer. Endogenous peroxidase blockade was performed with 3% hydrogen peroxidase, and nonspecific binding was inhibited. The sections were incubated overnight in Kiss-1 primary antibody (1:500, Millipore, # NP-002247) and then incubated with Alexa Flour 594 conjugate (1:800; Cell Signaling, USA, #anti-rabbit IgG [H + L]; red) for 1 h at room temperature. Afterwards, they were incubated overnight in GPR54 primary antibody (1:200; Novus, USA; #NLS1926) in the same sections and incubated with Alexa Fluor 488-conjugated streptavidin (1:300; Jackson ImmunoResearch, USA, #131489; green) for 1 h at room temperature. Finally, the sections were counterstained with DAPI to pinpoint the cell nucleus.

Confocal laser microscopy was performed at the excitation wavelengths of 488 and 594 nm, from which protein fluorescence was visualized. The location at ARC was analyzed using the third ventricle (interaural from 7.2 to 5.64 mm, bregma from − 1.8 to − 3.36 mm) [19]. Each section was collected in accordance with the same parameters. The position of ARC was first determined in a 200-fold field, and a 400-fold field was then used to select six fields of view imaging and for the quantification of the number of positively stained cells in each field of view.

### Statistical analysis

All data are presented as mean ± standard deviation. ANOVA was used to analyze the effects of age (PND 21, 43, and 56), exercise (exercise and sedentariness), and diet (+ and normal) on the biological indices in rats. Three-factor variance analysis was used to determine the presence of a three-factor interaction among independent variables, in which case a simple two-factor analysis was continued. Alternatively, if a two-factor interaction was observed, a simple independent-effect test was used. Statistical analysis was carried out on SPSS version 21.0 (SPSS Inc., Chicago, IL, USA). *P* < 0.05 was considered statistically significant.

## Results

### Changes in Kiss-1–GPR54 signaling pathway of growing rats

**Fig. 2:**
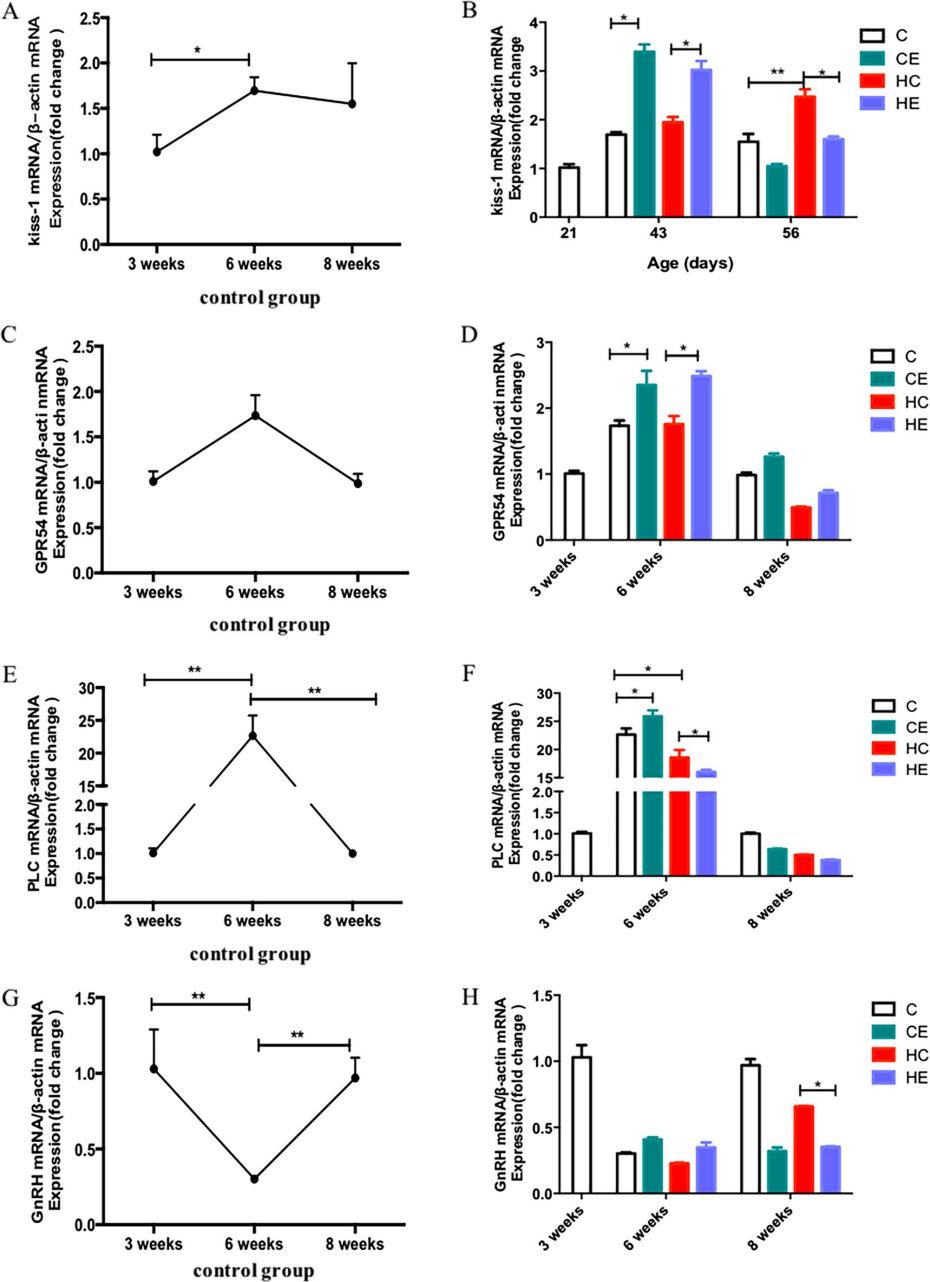
RT-qPCR analysis of Kiss-1, GPR54, PLC, and GnRH in the hypothalamus following high-fat diet and 60–70% $\dot{\mathrm V}{\mathrm O}_{2\max}$ treadmill training on PNDs 21, 43, and 56. **A** Kiss-1 in the control group on PNDs 21, 43, and 56. **B** Effects of high-fat diet and 60–70% $\dot{\mathrm V}{\mathrm O}_{2\max}$ treadmill training on the expression of Kiss-1 mRNA in rats on PNDs 43 and 56. **C** GPR54 in the control group on PNDs 21, 43, and 56. **D** Effects of high-fat diet and 60–70% $\dot{\mathrm V}{\mathrm O}_{2\max}$ treadmill training on the expression of GPR54 mRNA in rats on PNDs 43 and 56. **E** PLC in the control group at PNDs 21, 43, and 56. **F** Effects of high-fat diet and 60–70% $\dot{\mathrm V}{\mathrm O}_{2\max}$ treadmill training on the expression of PLC mRNA in rats on PND 43 and 56. Statistical analyses were performed by assessing three-factor variances (*n* = 6 per group; error bar, mean ± SEM.*, *P* < 0.05)

Kiss-1, GPR54, and PLC mRNA levels in the hypothalamus were measured to determine whether the Kiss-1–GPR54 signaling pathway was affected by age (P < 0.01). A similar variation tendency was found in Kiss-1, GPR54, and PLC mRNA in the hypothalamus during development. It fluctuated with the growth and development of male rats. All three genes first increased and then decreased from PND 21 to PND 56 and reached a peak at PND 43 (Figs. 2A, C, E, and 4A, C). When Kiss-1 protein expression in the ARC was analyzed via immunofluorescence. The results showed that the expression also reached a peak at PND 43, consistent with the results of mRNA (Fig. 4A). In contrast to Kiss-1 protein, GPR54 protein did not change during development (Fig. 4C). The number of cells expressing GPR54 protein was greater than those expressing Kiss-1 in the ARC throughout the development process (Supplementary Fig. 1A, 1B, 1F).

### Effect of high-fat diet on the expression of Kiss-1–GPR54 signaling pathway of growing rats

As shown in Fig. 2B, high-fat diet intervention upregulated the mRNA expression of Kiss-1 in the hypothalamus. Compared with the C group, the mRNA expression of Kiss-1 in the HC group showed a significant increase on PND 56 (Fig. 2B, *P* < 0.05). Furthermore, rats with high-fat diet increased the protein expression of kisspeptin in the ARC compared with the C group on PNDs 43 and 56 (Fig. 4B, *P* < 0.05).

PLC was downstream of the Kiss-1-GPR54 system, and it was a key factor in stimulating voltage-gated calcium channels. As shown in Fig. 2E and F, the expression of PLC mRNA on PND 43 was significantly higher than in other periods, and high-fat diet decreased the expression of PLC mRNA in the hypothalamus, especially on PND 43.

### Effect of exercise on the expression of Kiss-1–GPR54 signaling pathway of growing rats

**Fig. 3:**
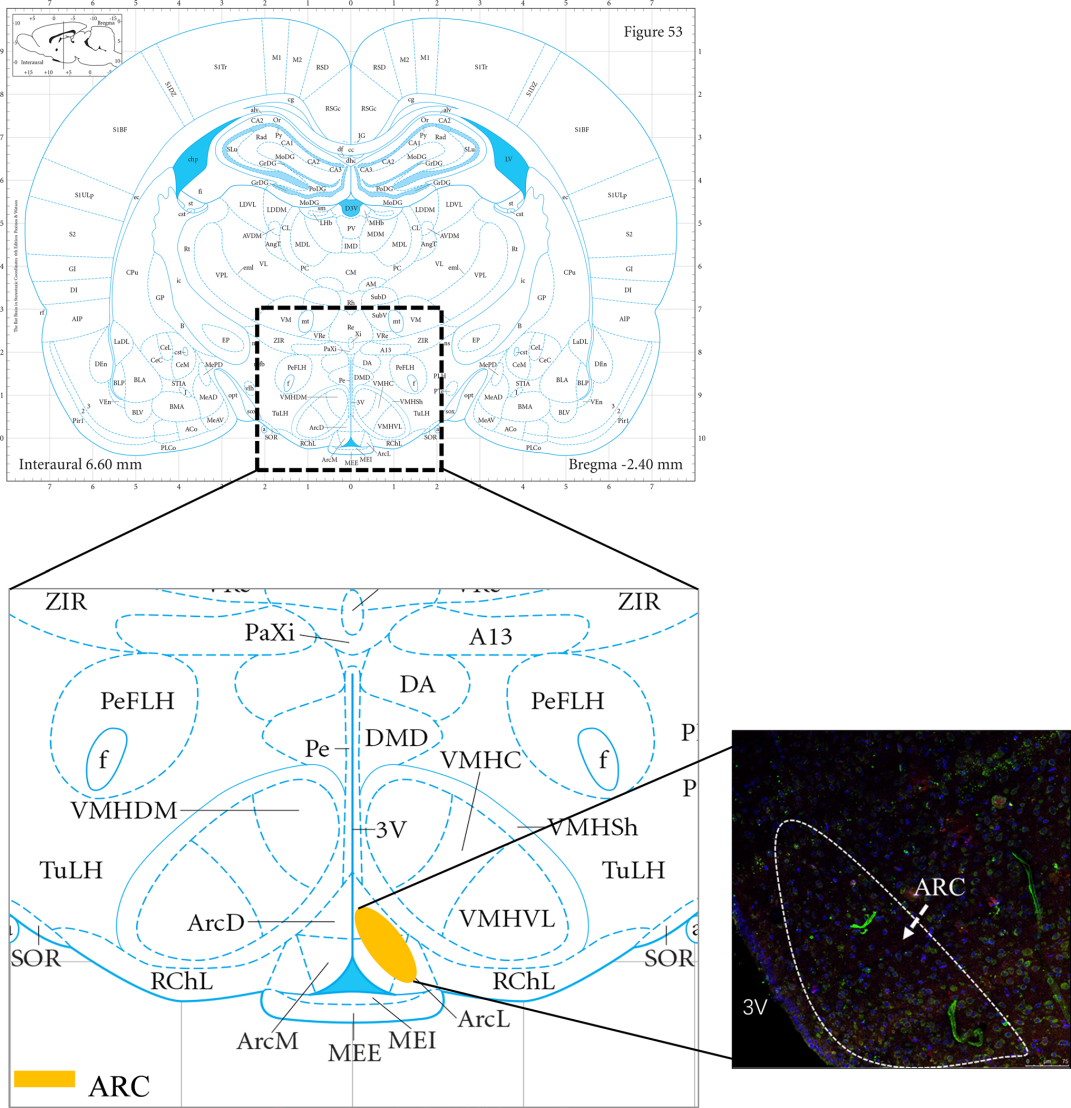
Brain in stereotaxic coordinates: the majority of kisspeptin and GPR54 neurons are located in ARC in the hypothalamus. ARC, arcuate nucleus; 3 V, third ventricle (Fig. 3 derived from 19)

**Fig. 4:**
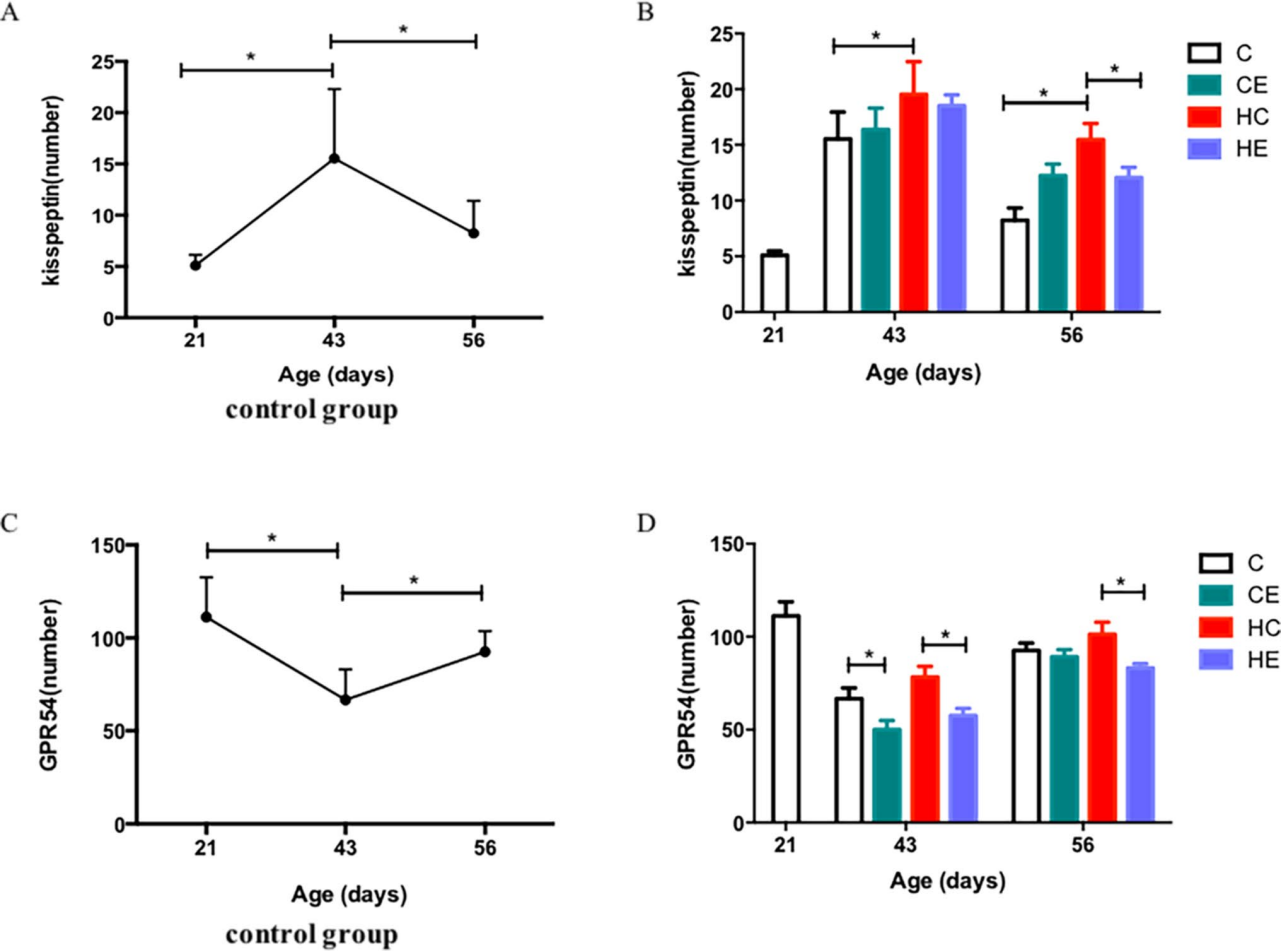
Immunofluorescent analysis in the hypothalamic ARC following high-fat diet and 60–70% $\dot{\mathrm V}{\mathrm O}_{2\max}$ treadmill training on PNDs 21, 43, and 56. **A** Kiss-1 antibody in the hypothalamic ARC of the control group on PNDs 21, 43, and 56. **B** Effects of high-fat diet and 60–70% $\dot{\mathrm V}{\mathrm O}_{2\max}$ treadmill training on the protein expression of Kiss-1 in rats on PNDs 43 and 56. **C** GPR54 antibody in the hypothalamic ARC of the control group on PNDs 21, 43, and 56. **D** Effects of high-fat diet and 60–70% $\dot{\mathrm V}{\mathrm O}_{2\max}$ treadmill training on the protein expression of GPR54 in rats on PNDs 43 and 56. Statistical analyses were performed by assessing three-factor variances (*n* = 3 per group; error bar, mean ± SEM.*, *P* < 0.05)

The expression of the Kiss-1–GPR54 signaling pathway on PNDs 43 and 56 in the CE and HE groups was examined to investigate whether the effect of exercise intervention on male rats of different ages on a high-fat diet were consistent. The results showed that the expression of Kiss-1 mRNA in the hypothalamus on PND 43 was significantly increased after continuous 60–70% $\dot{\mathrm V}{\mathrm O}_{2\max}$ treadmill training in high-fat diet rats (Fig. 2B, *P* < 0.05). The GPR54 mRNA levels of the hypothalamus in the CE and HE groups significantly increased after exercise intervention (Fig. 2D, *P* < 0.05). Exercise intervention was also able to upregulate the Kiss-1–GPR54 signaling pathway in high-fat diet rats on PND 43. However, among mature rats it showed a different result. As shown in Fig. 2B, exercise intervention downregulated the expression of Kiss-1 mRNA and protein in high-fat diet rats on PND 56, which was the maturity period (Figs. 2B, 3F-I, and 4B; *P* < 0.05). The expression of GPR54 protein in the ARC of the hypothalamus in the HE group was also remarkably lower than that in the HC group on PND 56 (Fig. 4D; *P* < 0.05).

### Effect of high-fat diet and exercise on body mass and reproductive system of male rats

**Fig. 5:**
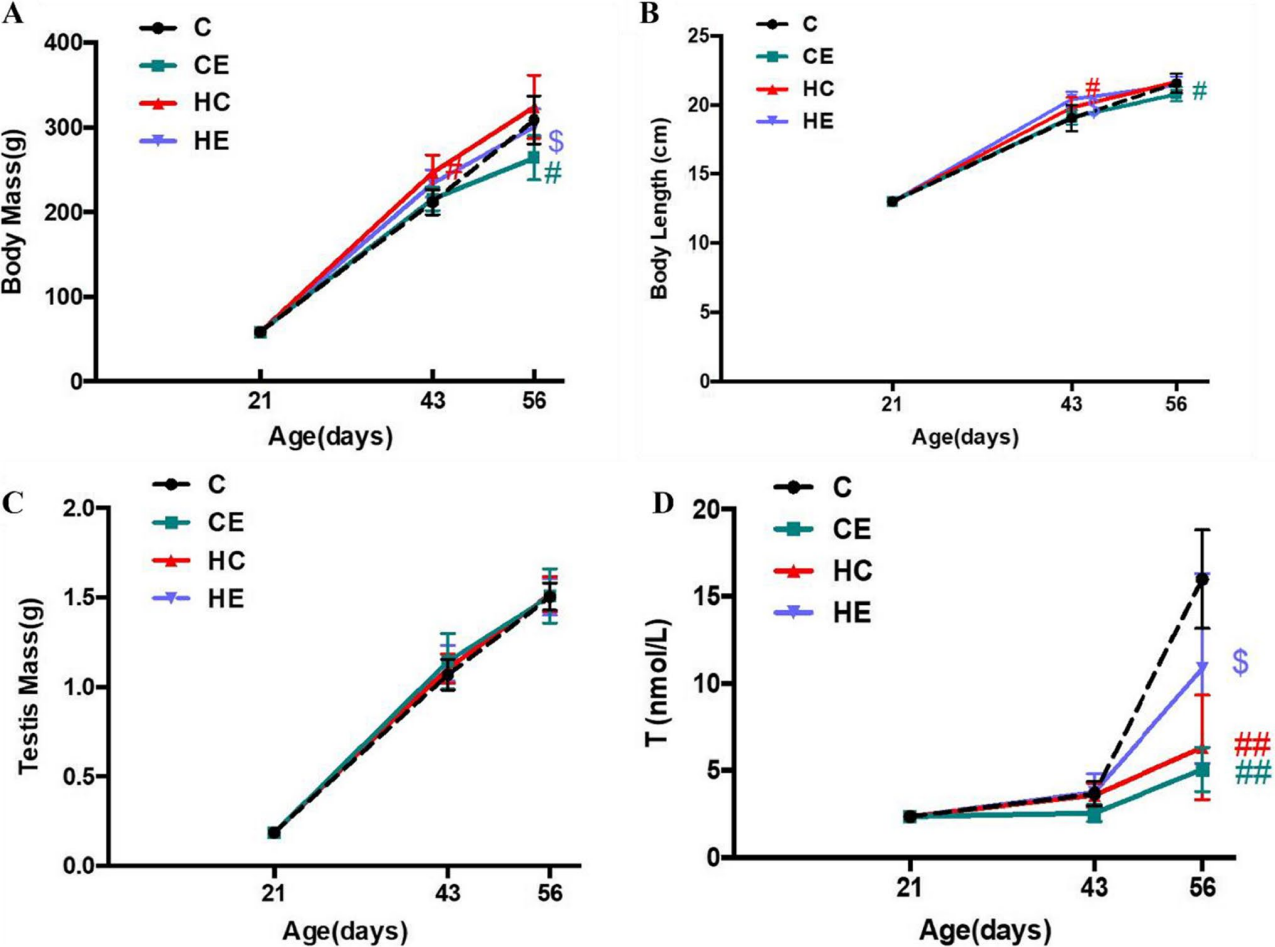
Effects of high-fat diet and 60–70% $$\dot{\mathrm V}{\mathrm O}_{2\max}$$ treadmill training on the body mass and reproductive hormones of rats on PNDs 21, 43, and 56. **A** Body mass changes on PNDs 21, 43, and 56. **B** Body length changes on PNDs 21, 43, and 56. **C** Testicular weight changes on PNDs 21, 43, and 56. **D** CLIAs for serum testosterone of rats on PNDs 21, 43, and 56. ^#^ *P* < 0.05 versus C, ^&^ *P* < 0.05 versus CE, ^$^ *P* < 0.05 versus HC

Body weight and body length increased gradually with growth in all groups. High-fat diet intervention significantly upregulated body mass and body length on PND 43 (*P* < 0.05, Fig. 5A and B) but not on PND 56 (*P* > 0.05). Exercise intervention significantly downregulated body mass both in standard-diet rats (*P* < 0.05) and high-fat diet rats (*P* < 0.05) on PND 56. In addition, body mass and length showed a tendency to increase after exercise intervention in rats on a standard diet on PND 43, consistent with the change in Kiss-1.

**Fig. 6:**
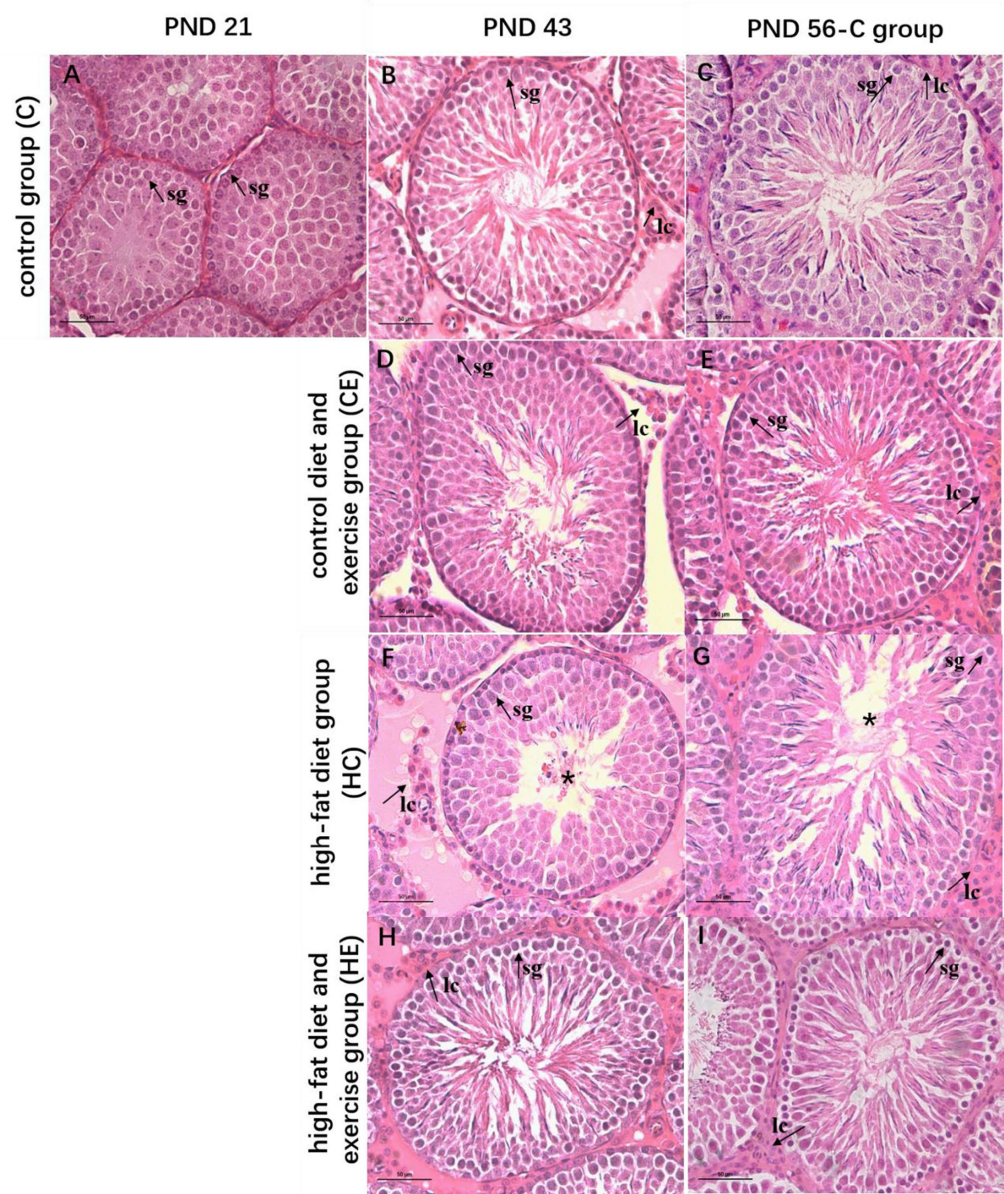
Effects of high-fat diet and 60–70% $\dot{\mathrm V}{\mathrm O}_{2\max}$ treadmill training on the seminiferous tubules of rats on PNDs 21, 43, and 56. **A**–**I** HE staining of testis in different groups stained with hematoxylin–eosin. sg—show examples of spermatogonia, lc—show examples of Leydig cells and stars—show absence of semen distribution in seminiferous tubules under × 200 magnification. (*n* = 6 per group, scale bar = 50 μm)

In the C group, testicular mass significantly increased with the increase in body mass and body length in all groups. The results of HE staining also showed that with the development of the testes, the number of sperm cells and spermatocytes increased in the C group at different age stages (Fig. 6A–C). Meanwhile, the serum testosterone levels increased at all stages of development from childhood to maturity as the morphology of testes matured, and they significantly increased on PND 56 (Fig. 5D, *P* < 0.05).

After high-fat diet or 60–70% $\dot{\mathrm V}{\mathrm O}_{2\max}$ treadmill training intervention, the weight of the testes did not show any change (Fig. 5C), but HE staining in high-fat diet rats showed that testicular lipid droplets increased, while the number of spermatozoa and Leydig cells in the testes decreased. The HE staining results demonstrated that the epithelium of spermatogenic cells was arranged loosely, and in the seminiferous tubules, vacuoles formed between spermatocytes and spermatoblasts due to the deposition of lipid droplets (Fig. 6F and G). Serum testosterone levels of high-fat diet rats decreased (*P* < 0.05, Fig. 5D), particularly on PND 43. Exercise intervention was able to increase serum testosterone levels of high-fat diet rats (Fig. 5D, *P* < 0.05), but it also reduced those of standard-diet rats, especially on PND 56. Meanwhile, the results of HE staining showed that the vacuoles formed between spermatocytes and spermatoblasts decreased with exercise, and the number of Leydig cells increased in the HE group compared with the HC group (Fig. 6H and I).

## Discussion

In this study, the growth and development of male rats and the parameters of the hypothalamic Kiss-1–GPR54 signaling pathway were measured at three timepoints, as follows: (a) PND 21 (early childhood); (b) PND 43 (puberty), and (c) PND 56 (maturity). Body mass, body length, testes weight, and serum testosterone gradually increased during development. The expression levels of the Kiss-1–GPR54 signaling pathway exhibited non-linear dynamics across growth. Kiss-1, GPR54, and their downstream factor PLC increased continuously from PND 21 and reached a peak on PND 43, and they decreased measurably on PND 56. Takumi et al. [20] measured the relative expression of Kiss-1 in the hypothalamus of rats at 3, 4, 5, 6, and 8 weeks old and found that Kiss-1 expression increased gradually from 3 weeks old to 5 weeks old and then decreased clearly at 8 weeks old, especially in female rats. The Kiss-1 change in the hypothalamus fluctuated with growing in male and female rats and peaked at puberty. The expression of Kiss-1 in the hypothalamus was closely related to reproductive function. In previous studies on the effect of high-fat diet and exercise on the testes of growing male rats, the gonadosomatic index (GSI), which represents the reproductive development of rats, was also shown to increase first and then decrease across development [21]. The change of Kiss-1 reported in the above study is consistent with the results of our present study across development. It is possible that Kiss-1 in the hypothalamus fluctuated during the growing period both in male and female rats. The reason for this discrepancy may be that the specific time of pubertal onset and the peak time of Kiss-1 expression is slightly different between males and females, the previous study having used female rats.

GPR54, as a receptor of Kiss-1, is a membrane-bound protein expressed on GnRH neurons, and Kiss-1 regulates GnRH through GPR54. In the current study, although the expression of GPR54 mRNA was consistent with that of Kiss-1 during development, the expression of GPR54 protein was shown to be higher than that of Kiss-1 protein in ARC. A series of reactions may promote transcription of the GPR54 gene into protein during growth, thus ensuring that Kiss-1 is able to adequately act on GPR54.

Increasing evidence has demonstrated that the onset of puberty was largely dependent on body mass rather than chronological age [10]. Energy status was commonly linked to the expression of Kiss-1 [22], whereas puberty was initiated by the Kiss-1–GPR54 system in the hypothalamus. Takumi et al. [8] suggested that a high-fat diet could induce obesity and precocious puberty in female rats, and that earlier puberty was more frequent in obese girls [23]. In the present study, a high-fat diet model rather than an obesity model was established to investigate the effects of high-fat diet on Kiss-1-related indices and metabolism in the hypothalamus of male rats. The rats were given a high-fat diet with 45% fat content starting on PND 21. This approach yielded an energy-imbalance model, in which a high-fat diet could induce positive energy balance and increase body mass and length significantly compared to normal diet rats, especially on PND 43. We hypothesized that this change might be accompanied by changes in the expression of Kiss-1 in male rats. Furthermore, we found high-fat diet intervention significantly increased the expression of Kiss-1 protein in the ARC in male rats on PNDs 43 and 56, indicating that a high-fat diet also affected the Kiss-1-GPR54 system in the hypothalamus of male rats. Energy status was commonly linked to altered the onset of puberty and reproductive impairment [22]. A high-fat diet was observed to induce obesity and precocious puberty in female rats [8] and earlier puberty in obese girls [23].

In the present study, high-fat diet intervention significantly increased the expression of Kiss-1 in the ARC in male rats on PNDs 43 and 56, indicating that the high-fat diet also affected the Kiss-1-GPR54 system in the hypothalamus of male rats. Moreover, the results showed the effect of high-fat diet, not only on puberty stage but also on maturity stage. Chantacha et al. [24] found that the serum kisspeptin of obese men was positively correlated with body weight, and serum kisspeptin levels were significantly increased in obese men. These findings indicated that the expression of Kiss-1 in the hypothalamus of males at maturity was possibly affected by positive energy balance, such as high-fat diet and obesity. Another interesting finding in the present study is that although no significant increase in body mass was observed in short-term high-fat diet intervention from PND 21 to PND 43, the expression of Kiss-1 in the hypothalamus also increased. The work of Ullah et al. in male mice supports the present finding, namely, that the high-fat diet induced precocious puberty even in mice with normal body weight [1]. Furthermore, although the expression of Kiss-1 increased after high-fat diet intervention, it still maintained the changing trend during the development process of male rats: in other words, it increased first and then decreased and peaked on PND 43; moreover, the GSI curve also did not change during development [21]. We demonstrate that testes mass and GSI did not show any change after the high-fat diet, but serum testosterone levels decreased and testicular morphology was affected; meanwhile, the lumen of tubules also showed absence of semen distribution in seminiferous tubules and the number of Leydig cells decreased in the HFD groups. The function of reproductive organs is regulated by the HPG axis, especially Leydig cells [25, 26]. The decrease in the number of Leydig cells is accompanied by a decrease in serum testosterone levels. Another study from our team also found that the Kiss-1-GPR54 system in testicular tissue was affected by a high-fat diet and it was significantly correlated with testosterone levels [21]. All these findings suggested that there is a close relationship between reproductive organs and Kiss-1 in the hypothalamus, a correlation which warrants further investigation.

Energy imbalance possibly induces the body to undergo a series of stress and/or adaptative responses. Matsuzaki et al. [14] reported that 72-h deprivation likely caused the expression of Kiss-1 mRNA to be significantly lower in female adult rats.

Similarly, True et al. [27] reported that 50% caloric restriction resulted in significant suppression of Kiss-1 mRNA in the anteroventral periventricular and ARC in adult female Wistar rats. The 60–70% $\dot{\mathrm V}{\mathrm O}_{2\max}$ treadmill training as a form of energy consumption could affect the male rats at different developmental stages; in the present study, we found that the effects of exercise intervention were different on PNDs 43 and 56. Most of these changes were found to occur at maturity since the expression of Kiss-1 mRNA, the protein amount of Kiss-1, and GPR54 in the HE group detected by immunofluorescent decreased after moderate-intensity exercise on PND 56 in high-fat diet group. The latter suggests that negative energy balance could inhibit the Kiss-1-GPR54 signal pathway both in male and female rats at maturity. Of note, moderate-intensity exercise induced a different result on PND 43: namely, we found that the exercise intervention decreased body mass, while it increased body length in male rats; meanwhile, the mRNA amount of Kiss-1 and GPR54 and the expression of Kiss-1 protein increased after exercise intervention. This was inconsistent with other negative energy balance conditions, such as undernutrition or fasting. A previous study demonstrated that calorie restriction evoked delayed reproductive development in prepubertal male rats [28]; this suggests that the changes caused by fasting are not the same as those caused by exercise. This distinction may be attributed to the fact that exercise is not only a means of regulating energy consumption but also a means of regulating metabolism, which could stimulate the secretion of hormones, such as growth hormones and sex hormones. Although the weight of rats decreased, their body lengths increased after exercise intervention. Increasing evidence has shown that puberty onset is largely dependent on body mass rather than chronological age [29, 30]. Moderate exercise may stimulate growth and development in puberty male rats and increase in the expression of the Kiss-1–GPR54 system. This feature is distinct from other regulators of negative energy balance, such as fasting, nutritional restriction, and cold environments.

In the present study, after exercise intervention, serum testosterone levels increased, and the lipid droplets in the testes of the HE group decreased on PNDs 43 and 56. In the work of Punhagui et al.[31], obesity having induced by high-fat diet caused damage to testicular functionality, resistance training could not reverse the sperm damage caused by obesity. However, the results of the present study show that in male rats on high-fat diet, semen distribution in seminiferous tubules was lower, and 60–70% $\dot{\mathrm V}{\mathrm O}_{2\max}$ treadmill training was able to reverse the change. Meanwhile, we also observed that exercise was able to reverse the reductions in Leydig cells and serum testosterone levels caused by the high-fat diet. A previous study also found that the serum testosterone concentration was lower in obese early-puberty boys and that aerobic exercise was able to increase the testosterone concentrations [32]. Leydig cells in the testes promote spermatogenesis and sperm transformation via testosterone synthesis, while Leydig cells and spermatogenesis concentrations rely on the production of LH and FSH from the anterior pituitary gland in response to GnRH from the hypothalamus [33]. These changes in the testes may be associated with the Kiss-1-GPR54 system. In addition, the infiltration of a high-fat environment could also induce Leydig cell apoptosis via the accumulation of oxidation products and the release of ceramide [34], which further aggravates the reduction in testosterone production induced by obesity. However, moderate-intensity exercise training could promote lipolysis and also reduce triglyceride and apoptotic cell numbers caused by high-fat diet [35]. All these findings showed that exercise intervention could correct the adverse effects of a high-fat diet on the hypothalamus and testes of male rats.

## Conclusions

- The Kiss-1–GPR54 signaling pathway in the hypothalamus of male rats fluctuated during development. It increased first, peaked at PND 43, and then decreased.
- A high-fat diet inhibited reproductive development and upregulated the expression of Kiss-1 in the hypothalamus in the growth phase of male rats.
- $\dot{\mathrm V}{\mathrm O}_{2\max}$ treadmill training (60–70%) was able to correct the adverse effect of high-fat diet on testicular function, upregulate the expression of Kiss-1 in the hypothalamus during puberty, and downregulate it at maturity.

## Supplementary Information

Below is the link to the electronic supplementary material.

Supplementary file1 (DOCX 2419 KB)
