# Supplementary material for: Effects of high-fat diet and treadmill running on the hypothalamic Kiss-1–GPR54 signaling pathway in male growing rats
Source: Hormones (Athens). 2022 Aug 24;21(4):641–52. doi: 10.1007/s42000-022-00389-4 (PMC9712360; doi:10.1007/s42000-022-00389-4)
Supplement: Supplementary file 1 — Supplementary file1 (DOCX 2419 KB) [file 42000_2022_389_MOESM1_ESM.docx]

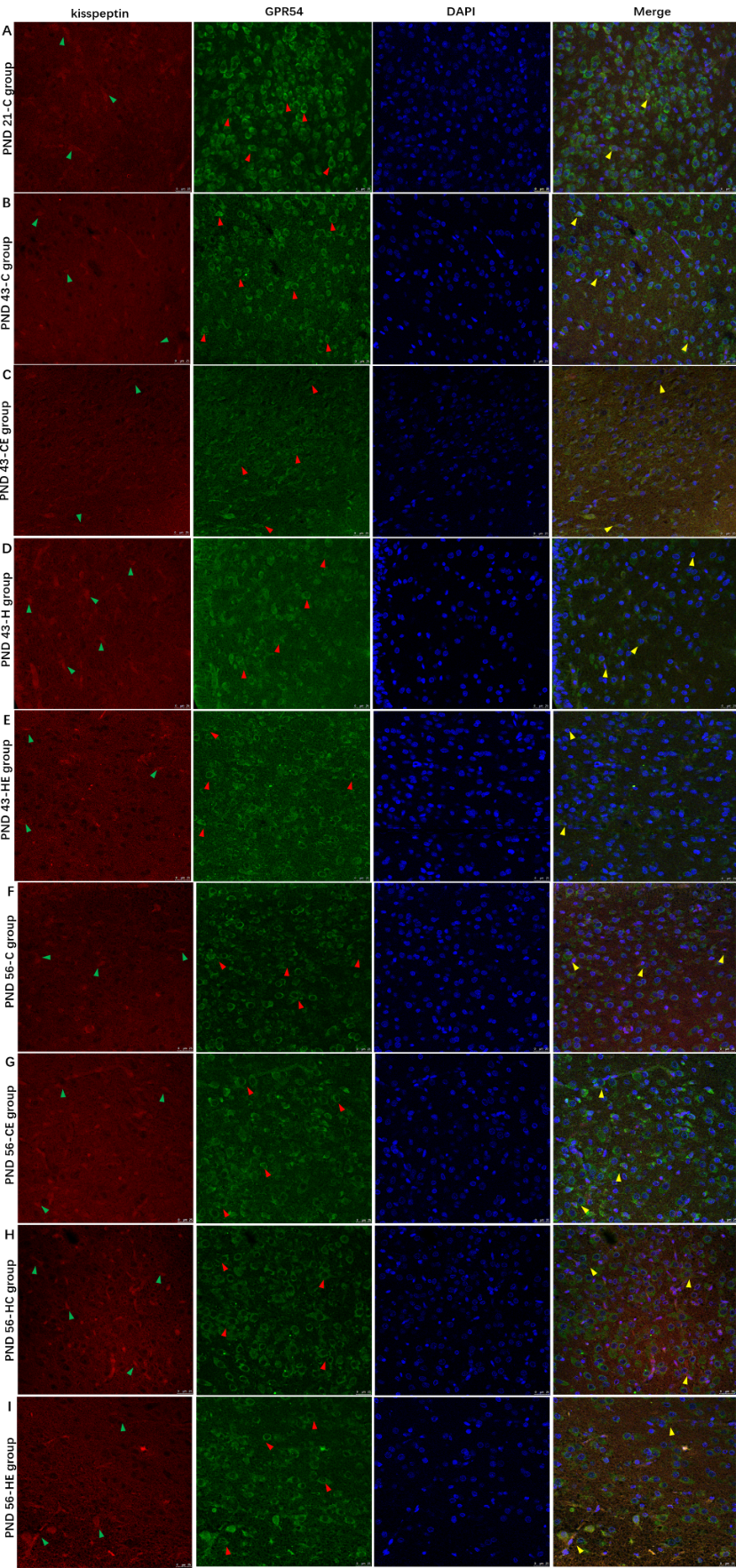
Supplementary material

Fig.S1 (A–I) Double-labelling Immunofluorescence results of Kisspeptin and GPR54 proteins in the ARC of hypothalamus following high-fat diet and 60%–70% O_2max_ treadmill training at PNDs 21, 43, and 56. The cell nucleus was stained by DAPI. Kiss-1 protein was labeled with red fluorescence (green arrows) and GPR54 was labelled with green fluorescence (red arrows). The yellow arrows indicate the merged cell nuclei. *n* = 3 per group, scale bar = 25 μm.
